# The response of Mesenchymal Stem Cells to endodontic materials

**DOI:** 10.1590/0103-6440202204786

**Published:** 2022-04-29

**Authors:** Patrícia Yanne de Oliveira, Mariane Floriano Lopes Santos Lacerda, Carlos Magno da Costa Maranduba, João Vitor Paes Rettore, Leda Quercia Vieira, Antônio Paulino Ribeiro

**Affiliations:** 1 Department of Operative Denstistry, School of Dentistry, Universidade Federal de Minas Gerais, Belo Horizonte, MG, Brazil;; 2 Department of Operative Denstistry, School of Dentistry, Universidade Federal de Juiz de Fora, Juiz de Fora, MG, Brazil;; 3 Department of Genetics, Institute of Biological Sciences, Universidade Federal de Juiz de Fora, Juiz de Fora, MG, Brazil;; 4 Department of Biochemistry and Immunology, Institute of Biological Sciences, Universidade Federal de Minas Gerais, Belo Horizonte, MG, Brazil;

**Keywords:** Mesenchymal stem cells, cytotoxicity, mineral trioxide aggregate, genotoxicity, dental pulp stem cells

## Abstract

An endodontic material must be minimally harmful to stem cells since they are essential, thanks to their capacity for cell proliferation, self-renewal, and differentiation. For this reason, in this in vitro study, the cell viability and the expression of genes involved in cell plasticity and differentiation were investigated in stem cells recovered from human dental pulp (hDPSCs) that were in contact with four endodontic materials (Endofill, MTA, Pulp Canal Sealer, and Sealer 26). The viability of HDPSCs was assessed by MTT and trypan blue exclusion assays. PCR evaluated cellular plasticity by determining the CD34, CD45, Nestin, CD105, Nanog, and OCT4 expressions. The effect on cell differentiation was determined by RT-PCR expression of the RUNX2, ALP, OC/BGLAP, and DMP1 genes. The data were analyzed using ANOVA with Bonferroni correction (p <0.05). Pulp Canal Sealer and Endofill decreased cell viability after 48 hours (p <0.001). MTA and Sealer 26 did not disrupt cell viability (p> 0.05). When cultivated in the presence of MTA and Sealer 26, hDPSCs expressed Nestin, CD105, NANOG, and OCT-4 and did not express CD34 and CD45. MTA and Sealer 26 interfered with DMP1, OC/BGLAP and RUNX2 expressions (p <0.05) but did not change ALP gene expression (p> 0.05). MTA and Sealer 26 showed biological compatibility in the presence of hDPSCs.

## Resumo

Um material endodôntico deve ser minimamente prejudicial às células-tronco, uma vez que essas células são extremamente importantes, devido à sua capacidade de proliferação, autorrenovação e diferenciação celular. Por esse motivo, a viabilidade celular e a expressão de genes envolvidos na plasticidade e diferenciação celular foram investigadas em células-tronco recuperadas de polpa dentária humana (HDPSCs) que estiveram em contato com quatro materiais endodônticos (Endofill, MTA, Pulp Canal Sealer e Sealer 26). A viabilidade das HDPSCs foi avaliada pelos ensaios MTT e de exclusão de azul de tripano. A plasticidade celular foi avaliada pela determinação das expressões dos genes CD34, CD45, Nestin, CD105, Nanog e OCT4 por PCR. O efeito na diferenciação celular foi determinado pela expressão dos genes RUNX2, ALP, OC/BGLAP e DMP1 por RT-PCR. Os dados foram analisados por ANOVA com correção de Bonferroni (p <0,05). Em comparação com o controle, Pulp Canal Sealer e Endofill diminuíram a viabilidade celular após 48 horas (p <0,001). MTA e Sealer 26 não interromperam a viabilidade celular (p> 0,05). Quando cultivado na presença de MTA e Sealer 26, as HDPSCs expressaram Nestin, CD105, NANOG e OCT-4 e não expressaram CD34 e CD45. MTA e Sealer 26 interferiram nas expressões de DMP1, OC / BGLAP e RUNX2 (p <0,05), mas não alteraram a expressão do gene ALP (p> 0,05). Sendo assim, MTA e Sealer 26 demonstraram compatibilidade biológica na presença de HDPSCs.

## Introduction

The endodontic treatment culminates with the complete filling of root canal systems that avoid microbial infection. Although sealers are expected to be confined within the root canal space, sealer's contact with periapical cells by apical foramen may occur, interfering in periapical responses and resulting in delayed wound healing ^1^. Hence, Endodontics has always required the use of materials well tolerated by apical tissues, presenting antimicrobial effects and promoting healing ^2^. Endodontic sealers based on zinc oxide and eugenol (ZOE) have been standard in endodontics since their development, based on their long-term success ^3^. For decades, Endofill (Dentsply Maillefer, Providência, Santiago, Chile) and Pulp Canal Sealer (Kerr Sybron Endo, Orange, California, USA) prevailed in the Brazilian and USA market, respectively ^3^, although ZOE may induce periapical inflammation ^4^. Moreover, seeking antimicrobial effects and stimulation of periapical tissues healing, root canal sealers based on calcium hydroxide became available in the late 1980s ^5^, such as Sealer 26 (Dentsply, Petrópolis, Rio de Janeiro, Brasil).

Another vital material incorporated in the endodontic arsenal was the Mineral Trioxide Aggregate (MTA; Angelus, Londrina, Paraná, Brazil), used as a dental root repair material and developed by Mahmoud Torabinejad in 1993 ^6^. MTA is indicated in pathological or iatrogenic root perforations ^6^ as well as in root-end fillings ^6^, but it has also been employed in pulp covering or pulpotomy ^7^. MTA is a powder composed of tricalcium silicate, bismuth oxide, dicalcium silicate, tricalcium aluminate, tetra calcium aluminoferrite, and calcium sulfate dihydrate ^8^. Due to its outstanding properties, nowadays, MTA is a gold standard in studies that compare the biological properties of dental materials ^8^.

Several parameters for testing endodontic sealers have been created ^9^ ^,^ ^10^. Studies have examined biological properties in many cells such as macrophages, fibroblasts, and endothelial cells ^4^ ^,^ ^8^ ^,^ ^11^ ^,^ ^12^. Moreover, analyses have demonstrated the importance of stem cells as a model to examine endodontic sealers' properties ^1^ ^,^ ^2^ ^,^ ^9^ ^,^ ^10^ ^,^ ^11^ ^,^ ^12^.

Stem cells (SCs) have been identified in many organs and tissues, and each type presents specific physiological properties. Hence, SCs recovered from dental pulp (HDPSCs) have become a great experimental model for studying the biological properties of dental materials since they are homologous to the cells that materials will be in contact with ^1^ ^,^ ^9^. Hence, mesenchymal stem cells (MSCs) have been demonstrated as an attractive cell source for tissue-engineering applications because of their ability to be easily isolated and expanded from dental pulp (HDPSCs) and their versatility for pluripotent differentiation into odontoblast and osteoblasts cells ^1^ ^,^ ^9^ ^,^ ^10^. However, few studies have chosen HDPSCs as a source in the endodontic material's behavior (1, 10), specifically on cell plasticity and differentiation. Then, this study aimed to investigate the effects of endodontic materials (Endofill, MTA, Pulp Canal Sealer, and Sealer 26) on these cells. For this purpose, cell viability, the expression of genes involved in cell plasticity, and cell differentiation were analyzed. The null hypothesis was that white MTA (Angelus) would not present any significant cytotoxic effects among all tested biomaterials, and then it could be selected as a control material.

## Materials and methods

### Stem Cells

The Genetic Laboratory of the Biological Sciences College of UFJF kindly provided HDPSCs. The Research Ethics Committee of the Federal University of Minas Gerais approved this study (CAAE- 87712218.9.0000.5149). HDPSCs were obtained from human exfoliated teeth after signing the Informed Consent Form by the donor (the donor's identity was kept confidential). The dental pulp processing was performed by mechanical tearing with the aid of a scalpel. The pulp was washed and cultivated in a basal medium consisting of DMEM/F12 medium (Invitrogen, Carlsbad, California, USA) supplemented with 10% (v/v) fetal bovine serum (FBS; LGC Biotechnology, Cotia, São Paulo, Brazil), 100 U/mL penicillin, and 100 µg/mL of streptomycin, 2 mM of L-glutamine and 0.01 mM of non-essential amino acids (Invitrogen, Carlsbad, California, USA) until the release and adherence of cells to the culture plate ^13^. The isolated HDPSCs were expanded and frozen in a freezing medium consisting of DMEM/F12 supplemented with 20% (v/v) of FBS (LGC Biotechnology, Cotia, São Paulo, Brazil) and 10% (v/v) of dimethylsulfoxide (DMSO; Sigma, Saint Louis, Missouri, USA). HDPSCs grew in 75 cm^2^ bottles. Cells were kept in 5% CO_2_ in the humidified atmosphere, at 37°C, until reaching a maximum confluence of 80% to 95%. Cell growth and morphology were monitored by transmitted light microscopy (Nikon TS100F, Tokyo, Japan). Analyzing cells' molecular profiles to infer chromosomal stability and consequent neoplastic potential, genes that act as markers of hematopoietic, mesenchymal, and embryonic stem cells as described elsewhere ^13^ were assayed in passage 5 of cells.

### Cell Culture

HDPSCs (1x106) were cultured in 5 mL of medium (D-MEM F12 medium; Sigma, Saint Louis, Missouri, USA), containing 10% (v/v) FBS (Nutricell, Campinas, São Paulo, Brazil), 2 mM L-glutamine, 100 mL units -1 penicillin, and 100 µg mL-1 streptomycin. Cells were placed in an incubator with a humidified atmosphere containing 5% CO_2_ at 37°C for 24 hours, allowing the cells to adhere to the bottom of the culture plate ^13^. The culture medium was changed at frequent intervals of 2 to 3 days until the cells reached 80 to 95% confluence, and the cells were used in passage 5.

### Endodontic Materials

All materials (Endofill, Dentsply; Sealer 26, Dentsply; MTA, Angelus; and Pulp Canal Sealer, Kerr Sybron Endo) were prepared following manufacturers’ instructions in sterile conditions (ultraviolet light was turned on for 30 minutes before all procedures). More information about the composition of each material evaluated in this study is presented in table 1. Soon after preparation, sealers were inserted into the tips of previously sectioned sterilized polyethylene capillary tubes (test group) so that their contact with the cell suspension could be standardized (Ø = 1.2 mm; length = 10 mm/area = 2.26mm2) ^8^. Empty capillary tubes were used in control cultures. Capillaries were sterilized by exposure to 25 kGray Gamma-ray irradiation (CDTN, Belo Horizonte, Minas Gerais, Brazil). All materials were left in an oven for 24 hours to set before all analyzes were carried out.

**Table 1:** The composition of endodontic materials

Endodontic Materials	Composition
Endofill (Dentsply, Maillefer, Chile)	Powder: Zinc Oxide, Hydrogenated Resin, Bismuth Subcarbonate, Barium Sulfate and Sodium Borate. Liquid: Eugenol, Almond Oil and BHT.
Pulp Canal Sealer (Kerr, Sybron Endo, USA),	Powder: Zinc Oxide, Precipitated Silver, Bismuth Subcarbonate, Barium Sulfate. Liquid: Oil of cloves, Balsam of Canada, Eugenol.
Sealer 26 (Dentsply, Petrópolis, Brasil),	Powder: Bismuth Trioxide, Calcium Hydroxide, Urotropin and Titanium Dioxide. Resin: Epoxy.
White MTA (Angelus, Paraná, Brasil),	Powder: tricalcium silicate, dicalcium silicate, tricalcium aluminate, calcium oxide and bismuth oxide. Liquid: distilled water.

### Cell Viability

HDPSCs were cultured for 48 hours in 96-well plates. Cell viability was determined by MTT (3-(4,5-dimethylthiazol-2-yl)-2,5-diphenyltetrazolium bromide) and trypan blue exclusion assays, as described elsewhere ^2^ ^,^ ^10^ ^,^ ^14^. The cytotoxicity tests were performed in triplicate and according to the ISO 10993-12:2012 (E). Analyzes were performed three times.

Growth curves were obtained through the MTT assay to establish the proliferation of stem cells. For analysis of the proliferation pattern, 1000 cells per well were seeded (day 0) in 96-well plates. The plates were divided into control and test groups so that the control group was cultivated in the absence of biomaterials, while Endofill, Sealer 26, MTA, and Pulp Canal Sealer influenced the test groups. For each group, 12-plicates were performed. After 24 and 48, the culture medium was removed, and a new culture medium was added with 10% of a previously prepared solution (5mg/ml) of the MTT reagent (Thiazole Blue Tetrazolium, code M2128, Sigma Aldrich. Saint Louis, Missouri, USA). Afterward, the plates were incubated in an oven at 37°C with 5% CO_2_ for 4 hours. The MTT medium was removed, and 200μL of the isopropanol-0.04M HCl acid solubilizer was added. The plates were incubated for one hour. The wells were read in the spectrophotometer (ELx800; Bio-Tek Instruments, Winooski, Vermont, USA) at 570nm using as white three wells with 200μL of the isopropanol-acid.

The integrity of the cell membrane and the direct count of the living and dead cells was evaluated by Trypan Blue ^14^. This dye does not enter living cells but passes through the membranes of dead cells via the sodium and potassium pump. Cell viability by trypan blue exclusion assay (GIBCO, Brazil) was performed in Petri dishes. The medium was removed from the wells, and cells were washed with 200μL of PBS. Cells were separated by the addition of 100μl of trypsin /EDTA 0.5%. RPMI-1640 supplemented with 10% FBS (50μL) and 0.5% trypan blue (50 μL) (Merck, Darmstadt, Germany) were added additionally to each well, and the plates were incubated for 5 minutes. Subsequently, a 20μL aliquot was removed and placed in a Neubauer Hemocytometer (Labor Optik GmbH, Germany). The number of viable and non-viable cells was finally counted under the microscope. At least 300 cells were counted per culture (performed in triplicate), and the results were expressed as a percentage of viability. The images were analyzed, in a double early, by previously calibrated researchers.

### Cell Plasticity

**Figure 1:**
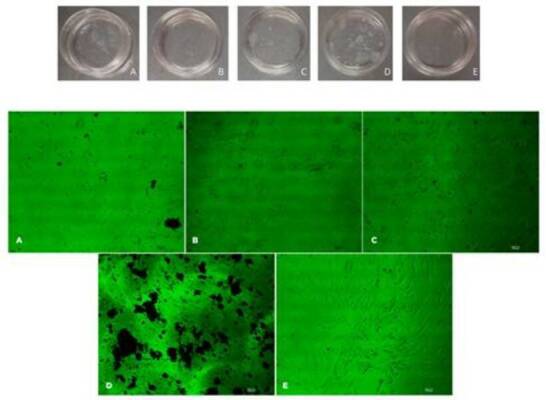
HDPSCs after culture and contact with endodontic materials. A (Endofill); B (Control); C (Pulp Canal Sealer); D (MTA); and E (Sealer 26). We can observe through microscopic images that in the presence of Endofill and Pulp Canal Sealer, hDPSCs cannot remain viable in sufficient quantities for RNA extraction and analysis of cellular plasticity. Bi-refractive structures are found in the cultures, suggesting that sealers might be dissipated from capillaries.

PCR analysis was used to assess genes that act as stem cell markers: CD34 and CD45 genes for hematopoietic cells; Nestin and CD105 for mesenchymal cells; and Nanog and OCT4 for embryonic stem cells. Beta-actin and GAPDH were used as the internal control. This investigation was restricted to HDPSCs cultures in which materials did not interfere with cell viability (Sealers 26 and MTA) so that a sufficient amount of RNA could be isolated for PCR analyses (Figure 1).

### Gene expression analysis

Total RNA from cultured cells was extracted using a reagent based on phenol and guanidine isothiocyanate (LGC Biotechnology, Cotia, São Paulo, Brazil) then; the RNA was resuspended in 50 μL of water treated with diethylpyrocarbonate (DEPC; Sigma Chemical Co., Saint Louis, Missouri, USA) containing 1 mM EDTA.

After extraction, total RNA was quantified by spectrophotometry (NanoDropTM 1000; Thermo Fisher Scientific, Wilmington, Delaware, USA), and all samples were diluted to a concentration of 2 μg/μL. According to the manufacturer's recommendations, cDNA was synthesized from total RNA using a High Capacity cDNA Reverse Transcription Kit (Thermo Fisher Scientific, Carlsbad, California, USA). The cDNA products were diluted in 87.5 μL of sterile water and used in PCR amplification experiments. The PCR mixture consisted of 2 μL of each sample and the following solutions: 0.8 μL of dNTPs (2.5 mM), 10 mM of TRIS-HCL pH 8.3, 50 mM KCl, 1.5 mM MgCl2, 0.6 μL of each primer, 0.05 μL (0.5 U) of Taq polymerase (GoTaq® Flexi DNA Polymerase, Promega Corporation, Madison. Wisconsin, USA), and 6.65 μL of sterile Milli-Q water. The polymerase chain reaction was carried out under standard conditions as follows: denaturation at 95°C (2 minutes), annealing for 40 cycles at 60°C (30 seconds) followed by 72°C (30 seconds), and 72°C (5 minutes). The sequences of the primers used in the PCR analysis of OCT4, NANOG, CD34, CD45, Nestin, and CD105 are shown in Table 2. The PCR products were separated by electrophoresis on 6% (p/v) polyacrylamide gels and then were visualized as bands by silver staining. The gel running condition was 80 v for 1h30min, analyzing the expected bands at the indicated heights. PCR analysis of each sample was performed two times.

**Table 2:** The sequence of primers for analysis of cell characterization.

Markers	***Primer* F**	***Primer* R**	*Amplicon (PB)*	Tm (°C)
Embryonic				
OCT4	ACTTCACTGCACTGTACTCCTCAG	AGGTTCTCTTTCCCTAGCTCCTC	158	60
NANOG	CTACCCCAGCCTTTACTCTTCCTAC	CTCTCCACAGTTATAGAAGGGACTG	217	60
Control				
Beta-actin	ATTAAGGAGAAGCTGTGCTACGTC	GATGGAGTTGAAGGTAGTTTCGTG	213	60
GAPDH	GAGTCAACGGATTTGGTCGT	TGGGATTTCCATTGATGACA	201	60
Hematopoietic				
CD34	AACACCTAGTACCCTTGGAAGTACC	AACACTGTGCTGATTACAGAGGTC	177	60
CD45	GGACACAGAAGTATTTGTGACAGG	GAGAAGTTGTGGTCTCTGAGAAGTC	176	60
Mesenchymal				
Nestin	GGACCCTCCTAGAGGCTGAG	GTGAGGAGAGGGGAGTAGGG	168	60
CD105	TGCCACTGGACACAGGATAA	CCTTCGAGACCTGGCTAGTG	205	60

### Cell Differentiation

Complementary DNA was synthesized using 1 μg of RNA and reverse transcribed as described previously ^15^. The primer sequences were designed using the Primer Express software (Applied Biosystems, Foster City, California, USA) based on nucleotide sequences available in the GenBank database. Real-time PCR assays were performed using the Primer Express software (Applied Biosystems, Waltham, Massachusetts, USA). The primer sequences used for the quantitative PCR analysis of RUNX2 (bone/tooth anabolic marker), BGLAP (OC) and ALP (osteoblasts), and DMP1 (an indicator of odontoblastic phenotype) are provided in Table 3. The PCR was performed under the following standard conditions: a holding stage at 95 °C (10 min); a cycling stage of 40 cycles at 95 °C (15 s), followed by 60 °C (1 min); and a melting curve stage at 95 °C (15 s), 60 °C (1 min), and 95 °C (15 s). An SYBR-Green detection system (Applied Biosystems, Waltham, Massachusetts, USA) was used to visualize primer amplification. Following amplification, a melting curve analysis was performed to determine the specificity of the amplified products. The melting curve was obtained from 60 °C to 95 °C, and continuous fluorescence measurements were recorded for every 1% increase in temperature. PCR products with melting temperatures that diverged from those established for standard DNA were considered false positives; for these cases, a null fluorescence value was attributed. HPRT1 and beta-actin were used as housekeeping genes for normalization and were assayed with each set of reactions. All samples were assayed in duplicate. Each reaction was performed in a 25-μL volume containing 1 μg of cDNA. The Sequence Detection System (SDS) Software version 2.4.1 (Applied Biosystems, Waltham, Massachusetts, USA) analyzed the data after amplification. The results were obtained as threshold cycle (Ct) values, and the expression levels were calculated using the comparative 2^-ΔΔCT^ method ^15^. The results were calculated as the mean value of duplicate assays for each patient. The mRNA expression levels in all samples were defined as the ratio of each specific primer to HPRT1 expression.

**Table 3:** The sequence of primers for analysis of cell differentiation.

Markers	***Primer* F e R**	***Melting* Temperature** **(ºc)**	Product Size	Fasta Pubmed Reference
HPRT1 ID 3251	F 5’ TGCTCGAGATGTGATGAAGG 3’ R 5’ TCCCCTGTTGACTGGTCATT 3’	54,5 56,1	192	NM_000194.2
B ID 60	F 5’AAACTGGAACGGTGAAGGTG 3’ R 5’GTGGACTTGGGAGAGGACTG 3’	55,4 57,1	206	NM_001101.3
* ALP ID 249	F 5’CCACGTCTTCACATTTGGTG 3’ R 5’AGACTGCGCCTGGTAGTTGT 3’	54,2 58,8	196	NM_000478.4
OC/ BGLAP ID 632	F 5’ GGCAGCGAGGTAGTGAAGAG 3’ R 5’ AGCAGAGCGACACCCTAGAC 3’	57,5 58,8	194	NM_199173.4
RUNX 2 ID 860	F 5’GAACTGGGCCCTTTTTCAGA 3’ R 5’CACTCTGGCTTTGGGAAGAG 3’	55,3 55,6	208	NM_004348.3
* DMP1 ID 1758	F 5’CAGGAGCACAGGAAAAGGAG 3’ R 5’CTGGTGGTATCTTGGGCACT 3’	55,6 56,9	213	NM_004407.3

*Galler et al., 2006

### Statistical analysis

Data analysis was performed using GraphPad Prisma software (version 7; GraphPad Software, Inc., San Diego, California, USA). The Kolmogorov-Smirnov test was used to verify normality and the Levene test for homogeneity of variance. The ANOVA test with Bonferroni correction was used to verify the statistical difference, followed by the Tukey test to verify the difference between the different materials. The significance level was 0.05.

## Results

### Cell Viability

**Figure 2:**
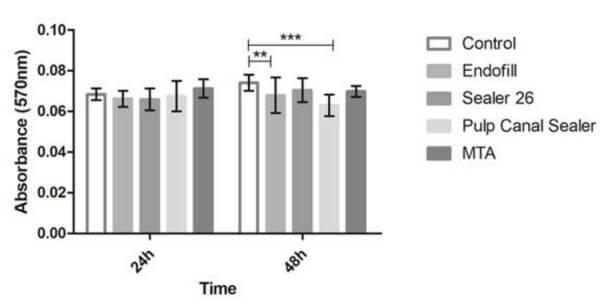
MTT assay of HDPSCs cultures at 24 and 48 h. Bars represent the average of the experiments; lines represent the standard error of the means. Values of p <0.05 are indicated by (*); p values <0.01 are indicated by (**); p values <0.001 are indicated by (***); and p values <0.0001 are indicated by (****).

Cell viability was assessed by MTT assay and showed that, compared to the control groups, no materials were cytotoxic after 24 h. However, at 48 h, Pulp Canal Sealer (Kerr Sybron) and Endofill (Dentsply) decreased cell viability significantly compared to the control group (p <0.001). MTA (Angelus) and Sealer 26 (Dentsply) did not affect cell viability in any of the conditions tested, indicating they were similar to the control groups (Figure 2). Trypan blue assay findings confirmed these results (data not shown). According to the ISO 10993-5:1999 (E) recommendations, biomaterials that promote a reduction in cell viability by more than 30% are considered cytotoxic. As Pulp Canal Sealer (Kerr Sybron) and Endofill (Dentsply) decreased cell viability almost entirely, cell plasticity and cell differentiation were not analyzed in the presence of both sealers.

### Cell Plasticity

**Figure 3:**
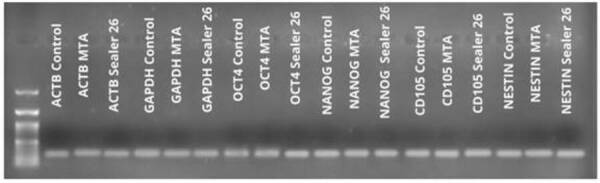
PCR amplification products were separated by electrophoresis on 6% (p/v) polyacrylamide gels and then were visualized as bands by silver staining. The tested markers are indicated. MTA and Sealer 26 positively expressed Nestin, CD105, NANOG, and OCT-4. PCR analysis of each sample was performed two times.

Cell plasticity was analyzed in the groups treated with MTA (Angelus) and Sealer 26 (Dentsply) for the reasons explained above. The gene expression levels of MSC markers Nestin and CD105 and the embryonic markers NANOG and OCT-4 were detected (Figure 3). However, gene expression of hematopoietic markers CD34 and CD45 was not detected in either culture group (data not shown).

### Cell Differentiation

**Figure 4:**
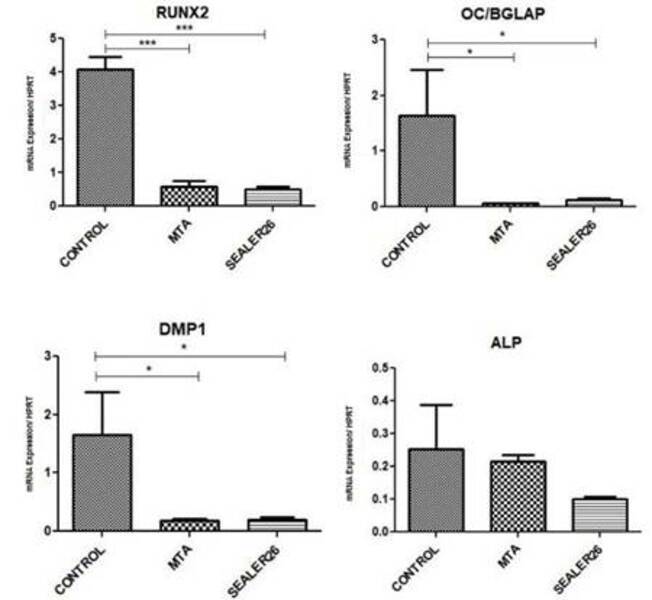
Gene expression of RUNX2, OC (BGLAP), DMP1, and ALP in HDPSCs. The Y-axis shows the values of mRNA expression relative to the expression of the endogenous controls. Values of p <0.05 are indicated by (*); p values <0.01 are indicated by (**); p values <0.001 are indicated by (***); and p values <0.0001 are indicated by (****).

MTA (Angelus) and Sealer 26 (Dentsply) significantly decreased the expression of DMP1, OC/BGLAP, and RUNX2 compared to their levels in the control group (p <0.05). Nevertheless, neither sealer interfered with ALP gene expression (p> 0.05) (Figure 4).

## Discussion

The biocompatibility of endodontic materials is of paramount importance, but it varies considerably and may cause adverse local effects due to the release of monomers or other organic and inorganic ingredients present in their composition ^3^. In this study, we used HDPSCs to assess the effects on viability and differentiation as well as the genotoxicity of four commercially available endodontic materials: Endofill (Dentsply), Sealer 26, (Dentsply), Pulp Canal Sealer, (Kerr Sybron), and MTA (Angelus). MTA was chosen as the control because of its well-demonstrated properties ^1^ ^,^ ^6^ ^,^ ^7^ ^,^ ^8^. White MTA presents any significant cytotoxic effects, a gold standard in this kind of experiment. Also, HDPSCs were a good choice since they differentiate into odontoblasts and osteoblasts cells, which endodontic materials will contact during clinical application ^1^. Furthermore, HDPSCs are easily obtained from human teeth ^1^ ^,^ ^13^.

Introduced in dentistry in the mid-90s ^6^, MTA presents excellent physicochemical composition and biological properties, promoting effective sealing, periodontal ligament repair and regeneration, bone recovery, and cementum formation ^8^ ^,^ ^16^. Moreover, MTA can be used in a humid environment without losing properties ^8^ ^,^ ^16^ ^,^ ^17^. Nowadays, MTA is the gold standard in biocompatibility assays ^1^ ^,^ ^6^ ^,^ ^7^ ^,^ ^8^. The tricalcium silicate is its main component ^10^ ^,^ ^17^. Due to its consistency and complex manipulation and insertion, their composition was improved, adding plasticizer in its composition ^17^.

The first strategy of this study was to evaluate the viability of HDPSCs when they were cultured in the presence of the materials. It was shown that Endofill (Dentsply) and the Pulp Canal Sealer (Kerr Sybron) decreased cell viability compared to that of control cells (p <0.001 and p <0.0001). Accordingly, similar results concerning Endofill in macrophage cultures have been previously demonstrated ^12^. Therefore, Pulp Canal Sealer impaired the viability of primary human cells recovered from periapical tissues and the animal lineage of fibroblasts and type I collagen ^2^ ^,^ ^11^. Conversely, in M1 and M2 macrophage cultures, Endofill and Pulp Canal Sealer impaired cell adherence and phagocytosis but did not interfere with cell viability ^18^. Previous studies attributed the cytotoxicity of endodontic cement based on zinc oxide and eugenol to the free eugenol released when handling the material ^4^ ^,^ ^12^. Its release is faster and more intense in humid environments, reaching toxic levels in the first moments of manipulation ^4^.

On the other hand, Sealer 26 (Dentsply) and MTA (Angelus) did not affect cell viability, similar to the control groups. In the literature, encouraging results concerning MTA have been reported regarding tissue tolerance and stimulation of mineralization ^8^ ^,^ ^16^. Conversely, it was observed that another type of MTA, the MTA Fillapex, reduced macrophage viability, adhesion, and phagocytic activity ^14^. Sealer 26 did not interfere with HDPSCs viability but reduced macrophage viability ^4^. Although Sealer 26 composition is based on bismuth trioxide, calcium hydroxide, urotropine, and titanic dioxide, along with epoxy resin ^5^, it did not contain Bisphenol-A, a mutagenic and cytotoxic component present in MTA Fillapex composition ^2^ ^,^ ^14^.

Following the cell viability tests, the second strategy of this study was to analyze the differentiation and genotoxic effects of the materials on HDPSCs, explicitly focusing on the materials that did not impair their viability, namely, Sealer 26 (Dentsply) and MTA (Angelus).

To be considered HDPSCs, cells must be isolated from human dental tissues, exhibit adherence capability, and fusiform morphology when adhered, contain the potential for differentiation into other cell types, and possess self-renewal ability ^13^. These cells positively expressed CD27, CD29, CD44, CD73, CD90, CD105, CD146, CD166, CD271, STRO-1, Nestin and Vimentin. In contrast, HDPSCs do not express CD34, CD45, CD14, or CD19, but they sometimes express embryonic cell markers, such as Oct-4, Nanog, and Sox-2 ^19^. Here, MTA (Angelus) and Sealer 26 (Dentsply) did not impair the expression of typical MSC markers, Nestin and CD105, or the embryonic markers NANOG and OCT-4. Moreover, neither material interfered with CD34 and CD45 gene expression (hematopoietic markers). Such findings demonstrate that neither material interfered with HDPSCs' cellular plasticity, validating them as excellent clinical materials. To our knowledge, such findings are unprecedented in the literature.

The genes involved in the cell differentiation process, RUNX2, ALP, BGLAP (OC), and DMP1, were evaluated in cells grown in the presence of materials. MTA (Angelus) and Sealer 26 (Dentsply) negatively interfered with the gene expression of DMP1, OC/BGLAP, and RUNX2 (p <0.05). As DMP1 induces the differentiation of immature dental pulp cells into odontoblasts ^20^, these data suggest that both sealers interfere with this crucial function.

RUNX2 is a transcription factor that controls the bone differentiation and maturation process by modifying the expression of several genes, such as OPN (SPP1) and OC (BGLAP) ^21^. RUNX2 drives pluripotent mesenchymal cells into the odontoblastic lineage ^22^. When RUNX2 expression is increased, osteoblast maturation is inhibited, OC expression is reduced, and OPN expression is increased ^25^. Here, RUNX2 expression was diminished by MTA (Angelus) and Sealer 26 (Dentsply), which favors the maturation of cells involved in tissue repair. Moreover, RUNX2 belongs to the Runt domain family, it is described as one of the most significant osteogenic transcription factors, and it is currently used as a marker of early osteogenic differentiation ^22^.

Alkaline phosphatase (ALP) analysis is essential for molecular biology and genetic engineering. ALP is responsible for cell proliferation and cell renewal in bone tissue and acts on odontoblasts to stimulate the proliferative process ^24^. MTA (Angelus) and Sealer 26 (Dentsply) did not negatively interfere with ALP gene expression, reinforcing the excellent biocompatibility of these materials.

Osteocalcin (OC) is a crucial component of bone, and it plays a role in bone mineralization and calcium homeostasis, being a significant indicator for the differentiation of osteoblast progenitor cells ^23^. It was observed that significant downregulation of OC in adipose-derived stem cells (ADSCs) drives the differentiation of osteoblasts ^23^. As MTA (Angelus) and Sealer 26 (Dentsply) negatively interfere with OC expression, both materials contribute to the regenerative processes. ALP, OC, and RUNX2 act in the processes of transformation or proliferation, maturation, and mineralization of the extracellular matrix ^24^, and here, it is observed that both sealers contribute positively to these processes.

This study aimed to analyze the effects of endodontic materials on the pluripotent plasticity and differentiation of HDPSCs since few studies have attempted to this subject ^1^ ^,^ ^10^. Despite the HDPSCs homogeneity, supporting patronization of temperature, pH, osmotic pressure, and CO_2_ levels, the in vitro limitation of this study, that performed assays in the specific times, deserves further studies to determine the toxicity of the materials in the long term. Moreover, the outcomes showed that Endofill (Dentsply) and Pulp Canal Sealer (Kerr Sybron) impaired HDPSCs viability. Conversely, MTA (Angelus) and Sealer 26 (Dentsply) did not interfere with the cell viability and the expression of markers involved in cell plasticity and cell differentiation. Moreover, it is interesting that, despite MTA (Angelus) different composition among the other materials and not being an endodontic sealer, it was selected as a control in this study based on its excellent performance described in the literature, which was also confirmed by the outcomes. Additionally, the results of this study stimulate further endodontic sealer studies to choose Sealer 26 as a standard control found in this MTA similar performance. Finally, the role of stimulated HDPSCs by the sealers in the pulp or periapical inflammation and the healing events remains debatable.
